# Chicago Dental Club—Memorial Meeting

**Published:** 1893-06

**Authors:** 


### CHICAGO DENTAL CLUB—MEMORIAL MEETING.

Regular meeting March 27, 1893, Dr. G. W. Haskins in the chair.

After the reading of the minutes of the previous meeting, the Club resolved itself into a memorial meeting and listened to speeches on the life and character of Dr. W. W. Allport.

The first speaker of the evening was Dr. L. P. Haskell, who said,—

Mr. Chairman, Walter W. Allport was born in Loraine, Jefferson County, New York, in June, 1824, so that had he lived until next June he would have been sixty-nine years old. He had a common-school education. Somehow his attention was called to the practice of medicine, and he entered the office of Professor Amasa Trowbridge, of Watertown, New York. I cannot say how long he remained; but he turned his attention to the practice of dentistry, entering the office of Dr. Robinson, of Watertown, where he remained several years, and then went to Rome, New York, and was associated with Dr. Perkins. In 1854 he removed to Chicago, and rented the corner of a room in a physician's office on Lake Street, just west of Clark, over the drug-store of J. N. Reed, the leading wholesale and retail house in the city, and where was kept for many years the only stock of dental goods.

My first acquaintance with Dr. Allport was in July, 1856. At that time his office was on Clark Street, opposite the court-house, in a two-and-a-half-story brick building with a basement. He had been here two years and had built up quite a practice, having secured the patronage of many of the best families, and was getting fees rather in advance of the times.

In 1858, Dr. Allport was elected President of the Western Dental Society. He was one of the orginators of the American Dental Association in 1860, and was elected its first chairman. In 1865 he was elected President of the American Dental Convention, and in 1886 was elected President of the American Dental Association.

In 1881 Rush Medical College conferred on him the degree of M.D., and he was for a number of years emeritus professor of dental surgery in that institution, and afterwards held the same position in the Chicago College of Dental Surgery.

He was instrumental in creating the dental section of the Ninth International Medical Congress, which met at Washington in 1887, and was made the Vice-President of the section, and labored assiduously to make it a success. He was one of the organizers of the

Chicago Microscopical Society, and for several years its President, and awakened much enthusiasm in microscopical research.

It was through his persistent efforts that the Columbian Dental Congress, which is to be held in Chicago next August, was organized, and to which the dentists of the world are invited, and of which he ought to have been elected President.

Dr. Allport was unique in his make-up as a dentist; in fact, I have often said it seemed as though he was "made to order" for an operator. A man of fine appearance, handsome, of commanding figure, pleasing in his manner, inspiring confidence in his patients, of keen perceptions and sound judgment, remarkably deft in the use of instruments, rapid in his movements, a mechanic and an artist; in fact, there seemed to be nothing lacking in qualifications for what he proved himself to be,—a dental operator without a peer. Not only this, but he was original in his methods. It was worth going a long distance to see him fill a cavity. Using but few instruments, and never at a loss as to the right one to lay his fingers on; going straight to the mark, making every stroke tell in the preparation of the cavity; then preparing his gold, he seized a pellet with the pliers, and passing it through the flame of the spirit-lamp, quickly put it in place, and holding the pliers between his teeth, rapidly packed the gold until the cavity was full. When polished, the product was a finished piece of work. As I remember him before the days of modern appliances,—engines, electric motors, rubber dams, mallets, etc.,—when the methods were far more difficult than now, twirling the burr in the fingers, using the file instead of the corundum and disk, the napkin instead of the dam, the hand-plugger instead of the mallet, and finishing-appliances crude as compared with the present ones, and yet seldom occupying more than an hour in filling the most difficult cavity, he was truly a master of his art. Just in the matter of changing napkins during the process of filling a lower tooth, with the saliva welling up, it was with a sleight of hand I never saw equalled.

I carried in my mouth for thirty-two years a gold filling in the posterior proximal cavity of an upper second molar, which could only be seen by the use of the mirror, which he was only fifteen minutes filling, and which was perfect when the tooth was extracted.

Some time in the sixties he commenced microscopical investigations as to the cause of the sensitiveness of dentine, the distribution of nerve-force in the tooth-structure. He conferred with Professor Leidy, of Philadelphia, who seemed to agree with him in his conclusions. About this time he performed a unique operation which excited much interest. It was the removal of a portion of the pulp in a molar tooth, dissecting a flap of periosteum, and covering the nerve, and then covering this with a temporary filling of gutta-percha. After waiting a certain length of time, he removed the filling and found a deposit of dentine over the pulp. This result he showed to many dentists from time to time for several years.

Dr. Allport was not only a fine operator, but he had fine taste in the arrangement of teeth for artificial dentures. In this he was unexcelled.

Dr. Allport was a good speaker, always able to express himself clearly and concisely, and equally also as a writer, as the papers he has written for the various societies and journals have shown. Among the earliest of these was a paper read in 1855, upon "Diseases of the Teeth," before the Cook County Medical Society, of which he was a member, giving the result of his investigations as to the effect of various acids upon the teeth. Later on was a valuable paper, which he read before the Boston Academy of Dental Science, in which he took strong ground in favor of a division of practice and educating a class of men exclusively in prosthetic dentistry. He had for many years labored assiduously in favor of the dental student being more thoroughly educated in medical knowledge; in fact, went so far as to propose that the dental student be educated in the medical college as a specialist, the same as the oculist, gynæcologist, etc., and then to go outside of the college to learn to make artificial dentures.

In 1863 and 1864 he edited the *People's Dental Journal*, a quarterly, whose object was to furnish the profession with reading matter pertaining to the teeth for the instruction of their patients.

Dr. Allport was not apt to be hasty in his judgment of men and matters, and would carefully weigh the subject, but when once his mind was made up it was difficult to swerve him. As is usually the case with men of strong and determined character, he made some bitter enemies as well as earnest friends. It was his ambition to do all in his power to elevate his profession, and so in concerting measures with this in view he often had contests with others. In doing this he usually carried his point, for he was shrewd and knew how to marshal his forces.

His family consists of his wife, daughter, and three sons. The oldest, Dr. Frank Allport, now practising in Minneapolis, has built up a lucrative practice as a specialist of the eye and ear. The second son, Dr. Walter H. Allport, is practising medicine in this city, and though yet a young man, has taken an advanced position in the profession. He is assistant surgeon at the Exposition grounds. The third son, Harry, is located in business at Portland, Oregon.

*Dr. Eugene S. Talbot.*—How often do we record in our books the death of some one of us who had spent many years in our profession! but who of us will leave behind the record of such an active life spent in dentistry as Dr. Allport?

Previous to my acquaintance with him in 1870, I looked upon him as a wonderful man. With the respect of the profession and people at large and his great ability, it seemed as if he possessed all the requirements for a successful practitioner. He was a disciple of the late Dr. Harris in believing that dentistry was a part of the great mother profession,—medicine,—and was instrumental in the establishment of the chair of dental and oral surgery in the seven medical colleges in Chicago; and also, that physicians might know something of the teeth and associate parts, he assisted in the formation of the Section of Dental and Oral Surgery in the American Medical Association. Since that time we have heard no more of the old subject, Is dentistry a specialty in medicine? That question has now been settled forever. Later he felt that many eminent dentists were prevented from becoming members of the Association because they did not hold the degree of M.D. He framed a resolution, which was offered by Dr. N. S. Davis, ex-President of the Association, which was adopted almost unanimously, there being only two dissenting voices, to the effect that graduates of such dental colleges that required a course of study equal to the best medical colleges should be admitted to membership in the Association; thus it seems that he has done all that was possible to draw the dental profession into the fold, and in 1882 he accomplished all that Dr. Harris dreamed and attempted in 1842.

My intimate acquaintance with him began in 1880, and I had always found him to be a man of integrity and honor, despising all that was low and seeking to elevate the profession. Strong in his likes and dislikes, and wholly unselfish, he was hated by many, but those who won his admiration can truly mourn for him. To the poor, struggling young student, just starting out on life's rough sea, he was a beacon-light, help in counsel and money coming from him when most needed and appreciated.

He was a firm friend and leader of the profession, always ready to put his shoulder to the wheel and help steer away from danger.

In conclusion, I can only say that the profession has been raised to a nobler standard by his having made it his life's work, and as the poet says,—

> " Gone before us, O our brother,
> To the spirit land!
> Vainly look we for another,
> In thy place to stand."

*Dr. E. L. Clifford.*—Mr. Chairman, to be called upon for an expression of respect, a tribute of affection in memory of a loved friend whose presence will never again lend light and dignity to our councils, is an honor and privilege I need scarcely say I appreciate.

When serious apprehensions were first expressed that Dr. Allport might not recover from his illness, I could not but recall the, to me, strange coincidence of my feelings of love and veneration for him, and, strange as it may seem to tell it here in Chicago, in which city I have lived for ten years only, my first recollections of the study of dentistry are fraught with reminiscences of Dr. Allport. When I was studying under my father away down in Louisiana, he exacted that I should listen with him to the reading of his dental journals by my mother. My mother always read his journals to him winter evenings. When a late journal was to be looked over, my father's first inquiry was, " See if there is anything this month from Dr. Allport;" consequently to me the name became a synonyme for authority upon the subjects upon which he wrote, and, under my father's direction as I grew older and advanced in my profession, I read everything I could find from his pen.

A few years after my graduation, fate sent me to Chicago, and certainly one of my most cherished wishes was to become acquainted, if possible, with Dr. Allport. Opportunity favored me at one of the meetings of our Dental Club, where for the first time I had the pleasure and honor of meeting him. I do not know why I say pleasure, for certainly I never met a man of whom I was more afraid and into whose presence I so much dreaded to come. I had read a paper that evening, and after the meeting Dr. Allport came to me and wanted to talk with me. When I tell you that in addition to my feeling of partial acquaintance with him through the media of dental journals, I found upon first sight of his venerable presence a striking resemblance to my revered father, you can, with me, possibly account for the magnetic influence of the man of years and wisdom upon the younger man, the anxious seeker after knowledge in fields of medical and dental lore. Dr. Allport's warm, cordial manner disarmed me. The fear of critic or censor vanished, our pleasant interview ended leaving with me the comfortable feeling of having gained a friend, and such time proved him to be, for in no emergency or dilemma did I ever find him indifferent to my solicitations or too busy to advise; and right here I shall quote his exact words when I felt, in duty to myself, bound to know how far or how often I might trespass upon his valuable time. (We all know his time over his chair was liberally paid for.) His reply was, "Dr. Clifford, I have been practising dentistry for nearly forty years, and I do not know that I have ever seen a day during that time that I did not have room enough on my appointment-books for another patient. There are practitioners who cannot take another patient, but I have never seen the time when I was not able to welcome a friend and glad to have him call on me. When you come to see me you will always receive a warm welcome."

Will you permit a brief simile? In the building up and beautifying of such cities as our own phenomenal Chicago many structures had to be removed, some demolished, and we younger men know that to many some of these were entitled to the distinction of being called landmarks. To the first settlers, the pioneers of our great city, landmarks were almost shrines, because around them clustered reminiscences of brighter, younger manhood, and though, with all the pride inherent to the progressive ambitious man, they saw them of necessity superseded by structures better fitted to the wants of an advancing age and population, yet the old were gone, and with them memories only cherished by those who had known them as rendezvous of important, serious deliberations or social relaxation, as the case might be, and when they met as neighbors and friends to discuss the work of those who, to them, were iconoclasts, the tribute of a sigh—maybe a tear—was offered to the relentless mandate of destiny. All things change; the old must give place to the new. In the rapid strides, the crowding needs of mental activity, the ambitions of professional life, the hurry, ay, and even the patient, hopeful toil of him who sees the topmost round of the ladder still hard to reach, learning, with each day's and each night's effort by the midnight lamp, that it is not reached at a bound. The lesson of the growth of our city is one we may to-night take home to ourselves in our professional lives, our hopes, our ambitions. Our honored predecessors, whose lives, whose attainments, whose example were not, as in the architectural world, "landmarks," but I will say beacon-lights, apostles of learning and wisdom,—shall we see them fall at our side and, the vacant chair filled by the younger, eager aspirant for name and place, refuse the tribute of a moment's pause from the routine of business, to say we honored, we loved them, and we miss them? I do not hesitate to say that I shall miss the storehouse of wise experience from which I was welcome to draw always, as was every other young progressive dentist. To me the death of Dr. Allport is indeed a personal loss professionally and socially, and I shall always regard it as one of the greatest honors of my professional life in Chicago to have known him, to have been associated with him in the same building, to have met his family, to have dined with him and had him dine with me, and last, though sad indeed the duty, to have been selected as one of the active pall-bearers at his funeral, the last effort that I could put forth for him.

*Dr. C. N. Johnson.*—I want to add my quota to what has been said regarding Dr. Allport. I became acquainted with him when I first came to Chicago in the fall of 1884. I called on him armed with a letter of introduction from Dr. Haskell, and the result of that call was that Dr. Allport gave a private clinic at his own office, the victim of the clinic being Dr. Haskell. Several others had operated on Dr. Haskell, and finally he said to me, "You fill that next tooth." I picked up the instrument, and the moment I did so, Dr. Allport made a remark that has inspired me ever since, that has encouraged me to do as good work as lay in my power.

I have been very intimately brought in contact with Dr. Allport recently on account of circumstances which I will relate. Last fall the editor of the *National Magazine*, a journal of American history, approached Dr. Allport for his biography, and asked him to select some one to write it. He selected me. In going over his past life and the struggles of his youth, I was brought into intimate relationship with him, and I will recite one incident which I think illustrates a phase of Dr. Allport's character that few of his friends know about. He was infinitely tender-hearted.

After I had finished the biography, before sending it to the publisher, I wanted to go over it with him to hear him verify the dates. I made an appointment with him to come to my office. He came, and I sat down and read the biography. In summing up the characteristics of the man, as any other biographer would do, I said something complimentary. It was nothing, however, but the truth. When I looked up, after reading it, I saw him sobbing like a child and the tears streaming down his face. It was a revelation to me, a complete surprise. After he went to his office he wrote me a letter of apology, as if a man needed to apologize for showing he had a tender heart!

In regard to Dr. Allport's ability, I saw something a short time since which was a monument to his skill. I was in his office and he showed me some fillings that he had put in over forty-two years ago. They were doing good service, with every prospect of lasting as much longer, if the patient lived. I might go on and mention many things regarding my personal experience with him; but personal reminiscences, however interesting to his friends, are scarcely adequate in dealing with a character like his.

This man whom we are called upon to mourn to-day started life at the bottom of a rough and rugged hill. A stone met his first step; a rock stood towering above his tiny form. But he turned his face resolutely towards the summit, and never lost sight of the star of hope. Thorns were in his path, ready to pierce his quivering flesh. Pebbles rolled beneath his feet. Storms swept down the mountain-side, and threatened to carry him into the depths of the valley below. But baring his breast to the blast, and lifting his brow towards the merest fleck of blue in the darkened canopy above, he never looked behind. When for a moment the force of adverse circumstances drove him struggling to the rear, he regained his lost ground by a burst of that magnificent courage which was his most conspicuous trait. His energies never flagged, his heart was never faint; and toiling on and on, through a lifetime of endeavor, he was at last rewarded by the attainment of a greater height than is given most men to reach. And standing there on the summit of the mountain's highest peak, his heroic form sharply outlined against the limpid blue, he paused for a moment,—a moment all too brief. Looking back over the field of his accomplishments, he saw scattered down the mountain-side the forms of many friends, and as the light began to dim his eyes, he waved his hand, in prophecy and adieu, and, turning, passed over into the limitless beyond.

We have nothing left but a memory, but so long as dentistry shall have a name the individuality of Walter Webb Allport will live beside it. And now, in this moment of final farewell, those who loved him best will mingle with those who loved him least, in weaving about his memory a wreath of immortelles.

*Dr. Ira B. Crissman.*—I had the pleasure of making Dr Allport's acquaintance at the organization of the Chicago Dental Club. Like Dr. Clifford, I always approached him with admiration, looking up to him with high esteem, seeming to feel my inferiority while in his presence.

I called on him when he was first taken ill, and found him very nervous, seemingly excited. After a lengthy interview, he concluded by saying, "Doctor, I have a few enemies. We cannot seem to come to an agreement. I would willingly lie down to-night and die if there could be an adjusted understanding between us." It was a very solemn utterance from one so highly honored and respected. I never had anything to impress me so deeply.

On the following Monday he sent for me, saying there was a tooth that was irritating him very much. I attended to the summons immediately; found the doctor in bed, being irritated by a sharp corner on a cuspid tooth, caused by abrasion. I removed the sharp edge by drawing a separating file over it. His mind seemed perfectly clear. The care and precaution he always observed in his every-day practice was not forgotten, even while suffering physical torture. He said, "Doctor, do not forget to wash your hands. You will find carbolic acid in that bottle on the wash-stand; remember, you cannot be too careful." He seemed to recognize the critical condition he was in, for on the following day when I called, he made this remark, "I'll be all right if the erysipelas does not go to my brain through my ear and nose." The next day he became unconscious. His life's candle was fast becoming extinguished. I looked upon him as a beacon-light, a light-house that stands on a hill only to be extinguished by death. His advice to me was always greatly appreciated. I shall always remember him as a senior guardian, one willing at all times to lend a helping hand to his many friends and associates. I feel that I will be a better man and do better work by having known him,—"a bright star in the professional firmament eclipsed."

*Dr. E. J. Perry.*—I simply wish to testify to the high character of Dr. Allport. While I have not known him as intimately as some of you, I have been drawn to him by the quality of his cordiality, affability, sincerity, gentleness, courage, high professional character, attainments, and venerable presence. He was a splendid man. I shall ever cherish his memory.

*Dr. A. E. Baldwin.*—Probably there is no member of the Chicago Dental Club who personally feels the loss of Dr. Allport more than myself. The years that I have known him have been, and will be as time goes by, fraught with the most pleasant memories. I came to Chicago entirely a stranger and a student in our specialty. Among the first that I met was Dr. Allport, and what I am to-day I owe more to him than to myself. These are his words when I first called upon him for counsel: "Dr. Baldwin, do not do as you contemplate doing. It won't pay to turn your back on any trouble. Face it and fight it out." I have lived only a few years since then, but I have learned that that was good advice. We all realize that a great man has gone from us,—a great man to the world at large in his influence and in his wide circle of acquaintance. It is somewhat surprising that he could find the time to be the help to me he was, and I find in talking with my friends that he also had time to devote to them and their interests. A man like Dr. Allport can do great and little things, and the little things have a greater influence in after-life than the great things. We can point to him as a professional man, as a friend of higher education, of advanced standards of learning, and it is a pleasure to us all to have been associated with him in any way.

A few years ago—you perhaps all know something of the circumstances—I was associated with Dr. Allport, by being an officer of the American Medical Association, in carrying out the formation of a section in the Ninth International Medical Congress, which met at Washington; I had a large correspondence in connection with that work, and was brought in more intimate contact with him. One time, while I was in his office, I said to him, "Dr. Allport, you are the greatest man I have ever met in the dental ranks." "Why?" he asked me. I said, "For this reason. You set your mark, and you can bring the event to pass that will bring success to it more than any man I ever saw. This matter is going to succeed, and largely through your efforts." He sat back in his chair, wiped his glasses, looked at me, and said, "Do you know why?" I made some remark, but I do not exactly remember the words. He said, "I have been a practitioner of dentistry for many years, and have been an observing man, and I have yet to be placed in a position where I did not advocate that which I firmly believed was right." Dr. Allport could grasp a point as quickly as any man I ever met; and he was not working for this or that office, he was working for what he assumed to be right, and in the instances where I went to him for advice, the first thing he wanted to grasp was the situation, and then his efforts would be directed towards doing thus and so, because he believed it was the right thing to do. I believe that Dr. Allport should be noted more especially for (1) his quickness of grasp, (2) his ability, and (3) his integrity and perseverance. There are very few men that I know of who would refuse the highest public office in connection with our specialty if it were offered them. I know that Dr. Allport did that, and others here know it. He had a reason for so doing. In a letter which he read to me regarding the matter, he said, "I would consider it a great honor and privilege to accept such an office, but I do not think it would be for the best interests of dentistry for me to do so. Another man [whom he named] would do better than I could do, because some see fit to misinterpret my motives and actions, and we must find some one upon which the profession can harmonize and work for this end." Is there anything grander than that a man can do? Is there anything higher he can do than that? If there is, it is beyond my comprehension.

A few weeks ago Dr. Allport was at my house, and he was feeling poorly, and I do not suppose I should have thought of this conversation if I had met him at the Society afterwards and he still alive and with us. A letter from Dr. Talbot a short time later stated that he was very ill, and probably would not recover. But I remember the conversation we had then. He spoke of his time on earth being very short. I do not think, however, he meant by it that he thought he was going to immediately die. He saw he was getting feeble, and while with me that day had to spend most of his time lying on a couch. He was having some trouble, of which we are all aware, and it was bearing heavily upon his mind, for the reason that he did not know what was exactly right, and he wanted to do what was right. In the remark he made to me in regard to the trouble he was having, he said, "I wish I knew what was the right thing to do." We all know that if Dr. Allport knew what was the right thing to do he would do it.

Dr. Johnson has said a thing that ought to be emphasized,—namely, that he had a tender heart, and was more conscious and careful of the feelings of others and his influence upon them than we can yet realize. Twenty years from now, if we are living, we can understand this man's life better than we can to-day. It has been remarked that Dr. Allport had enemies. I believe he had, but he had fewer enemies than we think. There are those that had axes to grind, who had political ends to attain, who knew that Dr. Allport was a stumbling-block in the way of getting these ends accomplished; and although he perhaps was disliked by some, I believe there were very few who did not in their hearts have the the utmost respect and veneration for him. I shall prize his memory and the fact of such an intimate personal acquaintance with him. I shall always feel that I am a better man for having met and known Dr. Walter Webb Allport.

*Dr. M. A. Newman.*—I think Dr. Allport was one of the few men whose words, written or spoken, upon subjects pertaining to dental science were always worthy the attention of the profession.

*Dr. I. A. Freeman.*—I am probably one of the oldest members present who has known Dr. Allport, and what I shall say with reference to him will be from the stand-point of a student and later as a practitioner. He was certainly at the head of the dental profession in this city at that time, although there were other dentists as highly respected. Every dentist always held Dr. Allport in high esteem. There were a few young men here, and they were all anxious to become familiar with his method of manipulating gold and the treatment of teeth. Every young dentist in practice for the succeeding fifteen years met with many of Dr. Allport's patients, who had left him because of his high prices, and in that manner we all came to know the character of his operations.

In his earlier as well as later years he was advanced upon the subject of treatment of teeth and of the filling of root-canals. I recollect his remarks at a meeting of the American Dental Association in Crosby's Opera-House, in 1865, where he outlined his process of treatment of diseased roots that had lost their crowns, and upon which he desired to place an artificial crown. At the same meeting he advocated the use of the rubber dam introduced by Dr. Barnum. While I always felt it desirable to make the acquaintance of a man who had attained the position he had, it was not my fortune to meet him personally until within the last ten years. As we know, there has been an antagonistic feeling on the part of many members of the profession towards him, but it is not because of his being in the wrong; he has been in the right largely. Dr. Allport has done much to help us in this better, broader, and more helpful condition of things. Every dental student, old or young, is to-day indebted to him for the stimulus he ever gave to higher dental education; the benefits the public are receiving at their hands is an ever-living testimony to his memory.

RESOLUTIONS OF THE CHICAGO DENTAL CLUB IN MEMORY OF DR. W. W. ALLPORT.

WHEREAS, In view of the loss we have sustained by the decease of our friend and associate, Dr. W. W. Allport, and of the still greater loss sustained by those who were nearest and dearest to him; therefore, be it

*Resolved*, That Dr. Allport was a man of good principles, lofty in his ideas, conscientious in his dealings, strong in his purpose for right, and far-sighted in the requirements of the profession, and that it is but a just tribute to the memory of the departed to say that, expressing our regret for his removal, we, the members of the Chicago Dental Club, mourn for one who was in all respects entitled to the regard and admiration of every one with whom he came in contact.

*Resolved*, That we sincerely condole with the family of the deceased on the dispensation with which it has pleased Divine Providence to afflict them, and commend them for consolation to Him who orders all things for the best, and whose chastisements are administered in mercy.

*Resolved*, That this heartfelt testimonial of our sympathy and sorrow be forwarded to the family of our departed friend,

L. P. Haskell,
E. S. Talbot,
C. N. Johnson,
*Committee.*

